# DNA Methylation Signature in Monozygotic Twins Discordant for Psoriatic Disease

**DOI:** 10.3389/fcell.2021.778677

**Published:** 2021-11-24

**Authors:** Matteo Vecellio, Elvezia Maria Paraboschi, Angela Ceribelli, Natasa Isailovic, Francesca Motta, Giulia Cardamone, Michela Robusto, Rosanna Asselta, Sonia Brescianini, Francesco Sacrini, Antonio Costanzo, Maria De Santis, Maria Antonietta Stazi, Stefano Duga, Carlo Selmi

**Affiliations:** ^1^ Division of Rheumatology and Clinical Immunology, Humanitas Research Hospital IRCCS, Rozzano, Italy; ^2^ Nuffield Department of Orthopaedics, Rheumatology and Musculoskeletal Sciences, University of Oxford, Oxford, United Kingdom; ^3^ Department of Biomedical Sciences, Humanitas University, Rozzano, Italy; ^4^ IRCCS Humanitas Research Hospital, Rozzano, Italy; ^5^ Italian Twin Registry, Centre for Behavioural Sciences and Mental Health, Italian National Institute of Health, Rome, Italy; ^6^ Dermatology, Humanitas Clinical and Research Center-IRCCS, Rozzano, Italy

**Keywords:** DNA methylation, twins, psoriartic arthritis, psoriatic disease, transcriptomic (RNA-seq), epigenetics (DNA methylation)

## Abstract

**Background:** Psoriatic disease is a multifactorial inflammatory condition spanning from skin and nail psoriasis (Pso) to spine and joint involvement characterizing psoriatic arthritis (PsA). Monozygotic twins provide a model to investigate genetic, early life environmental exposure and stochastic influences to complex diseases, mainly mediated by epigenetics.

**Methods:** We performed a genome-wide DNA methylation study on whole blood of monozygotic twins from 7 pairs discordant for Pso/PsA using the Infinium Methylation EPIC array (Illumina). MeDiP—qPCR was used to confirm specific signals. Data were replicated in an independent cohort of seven patients with Pso/PsA and 3 healthy controls. Transcriptomic profiling was performed by RNAsequence on the same 7 monozygotic twin pairs.

**Results:** We identified 2,564 differentially methylated positions between psoriatic disease and controls, corresponding to 1,703 genes, 59% within gene bodies. There were 19 regions with at least two DMPs within 1 kb of distance and significant within-pair Δ*β*-values (*p* < 0.005), among them SNX25, BRG1 and SMAD3 genes, all involved in TGF-β signaling pathway, were identified. Co-expression analyses on transcriptome data identified IL-6/JAK/STAT3 and TNF-α pathways as important signaling axes involved in the disease, and they also suggested an altered glucose metabolism in patients’ immune cells, characteristic of pro-inflammatory T lymphocytes.

**Conclusion:** The study suggests the presence of an epigenetic signature in affected individuals, pointing to genes involved in immunological and inflammatory responses. This result is also supported by transcriptome data, that altogether suggest a higher activation state of the immune system, that could promote the disease status.

## Introduction

Psoriatic arthritis (PsA) and psoriasis (Pso) represent different manifestations of the same disease spectrum, i.e., psoriatic disease, a systemic chronic inflammatory condition, where genetic factors contribute to disease susceptibility and where biomarkers are virtually absent (Ritchlin et al., 2017). Monozygotic twins (MZ) exhibit a variable degree of concordance for several complex disorders, including autoimmune diseases (Ceribelli and Selmi, 2020), and twin studies contributed to identify susceptibility genes. MZ twins show concordance rates as high as 64% for psoriasis, while in the case of PsA these are almost equal in MZ and dizygotic (DZ) twins although these rates are difficult to compare, based on the bias related to the coexistence of Pso and PsA (Pedersen et al., 2008). Nonetheless, these observations suggest that epigenetic factors might play a major role (Ceribelli and Selmi, 2020), as supported by the experimental evidence in healthy MZ twins (Brodin et al., 2015; Generali et al., 2017) and by the phenotype changes according to age in MZ twins (Fraga et al., 2005). Further, DNA methylation patterns are associated with MZ twins’ discordance in the imprinted regions of monogenic syndromes (Ollikainen and Craig, 2011) and in inflammatory diseases with largely incomplete MZ twin concordance, such as systemic lupus erythematosus, type-1 diabetes, rheumatoid arthritis, and systemic sclerosis (Absher et al., 2013; Coit et al., 2013; Liu et al., 2013; Ramos et al., 2019). Limited data from epigenome-wide association studies (EWAS) are available for Pso and PsA and a deregulated epigenetic machinery has been demonstrated in psoriatic skin and whole blood (Zhou et al., 2016).

We hypothesize that epigenetic changes, in specific DNA methylation, may contribute to the onset of Pso/PsA in discordant MZ twins. (Pai et al., 2015; Schübeler, 2015). We performed a peripheral blood EWAS on a cohort of 14 (seven pairs) MZ twins clinically discordant for Pso/PsA and identified DNA methylation changes with specific differentially methylated positions (DMPs) associated with psoriatic disorder. The characterization of a possible circulating psoriatic disease-associated methylome would identify specific candidate *loci* to be further investigated as molecular biomarkers.

## Materials and Methods

### Study Participants

We enrolled pairs of MZ twins previously identified through the collaboration with the Italian Twin Registry—Istituto Superiore di Sanità and residing in the area surrounding the Humanitas Research Hospital in Rozzano (Milan, Italy). Participants were evaluated jointly by a rheumatologist and a dermatologist to define the diagnosis of Pso according to the physical examination and/or PsA according to the CASPAR classification criteria (Taylor et al., 2006). The study was approved by the local IRB and, following the signing of an informed consent, all participants provided peripheral whole blood samples at the time of the joint rheumatological and dermatological evaluations. Whole blood and peripheral blood mononuclear cells (PBMCs) were obtained from both twins at the same time using heparinised vacutainers and processed in parallel. In total, 7 MZ twin pairs discordant for Pso or PsA (the presence of either being defined psoriatic disease) were included. Epigenetic data were validated in an independent cohort of seven patients with Pso/PsA and 3 healthy controls. Disease subtype, current and/or past drug history of the affected twins, comorbidities, family history, smoking status and smoking behaviour, diet, alcohol consumption and allergies of both twins were recorded.

### DNA Extraction and Methylation Analysis

Genomic DNA was extracted from whole blood using an automated DNA extractor (Chemagic Star workstation; Hamilton, ON, Canada), following manufacturer’s instructions. For each sample, 500 ng of genomic DNA was bisulfite converted using the EZ DNA Methylation Kit (ZymoResearch, Irvine, CA, United States). Bisulfite-treated samples were then processed using the EPIC array (Illumina, San Diego, CA, United States), according to manufacturer’s instructions. The iScan system (Illumina) was used to scan the arrays and obtain the raw intensity data files. DNA methylation data were processed using the R package minfi (Aryee et al., 2014), to obtain DMPs. First, a quality control was performed to evaluate sample-specific methylation parameters, exploiting the presence of control probes on the EPIC array. Then, the mean detection *p*-value was evaluated across all samples, and those probes with a *p*-value > 0.01 in one or more samples were discarded from the dataset. We also removed those probes where SNPs may affect the CpG, and underperforming probes, as suggested by Zhou and others (Zhou et al., 2017). Finally, we also filtered out probes localized on the X and Y chromosome, to avoid sex-specific effects. After this QC step, the final dataset included 762,451 probes, which accounts for 88% of the EPIC probes. Finally, data were normalized using the minfi quantile normalization algorithm. DMPs were calculated using the R-package limma (Ritchie et al., 2015), the function lmFit, and eBayes, based on a paired statistics, and correcting for batch effects with the sva package (Leek et al., 2020).

### Regulatory Annotations

The eFORGE v2.0 (https://eforge.altiusinstitute.org/) (Breeze et al., 2016) tool was used to identify if the associated CpGs (top 1,000) were enriched in cell-specific regulatory elements, such as DNase I hypersensitive sites (DHSs) (active regulatory regions) and loci with overlapping histone modifications (H3K4me1, H3K4me3, H3K9me3, H3K27me3, and H3K36me3) across available cell lines and tissues from the Roadmap Epigenomics Project, BLUEPRINT Epigenome, and ENCODE (Encyclopedia of DNA Elements) consortia data. Chromatin Hidden Markov Models (ChromHMMs) bioinformatic approach was adopted to annotate Pso/PsA-associated DMPs through the combination of epigenetic maps across multiple cell types (Chen et al., 2013).

### Gene Ontology Analysis

Enrichment analysis for the host genes of the differentially methylated positions was performed using the “topGO” Bioconductor tool, the “biological processes,” the “molecular functions” database, and the *elim* algorithm (Alexa et al., 2006). Gene ontology enrichment was performed on those genes containing at least two DMPs.

### MeDIP—qPCR

MeDIP was performed to detect immunoprecipitated methylated DNA with an anti-5′-methyl-cytosine antibody. The MagMeDIP qPCR kit (Diagenode, Belgium) was used following manufacturer’s instructions. Briefly, genomic DNA was sheared using a BioRuptor sonicator (Diagenode, Belgium) to produce 400 bp fragments, which were checked by gel electrophoresis. The obtained fragments were immunocaptured with a monoclonal antibody specific for 5-methyl cytosine (supplied by the kit). Methylated DNA was then washed and purified from beads with a DNA Isolation Buffer (DIB) and proteinase K provided by the kit. Following an incubation at 55°C and at 100°C (for 15 min each), the supernatant containing the DNA was used for qPCR analysis to evaluate enrichment.

### RNA Sequencing

Whole blood was collected in a PAXgene Blood RNA Tube (PreAnalytiX, Switzerland) and RNA extraction performed using the Maxwell simplyRNA Blood Kit (Promega, United States), following the manufacturer’s instructions. RNA quality was assessed by LabChip GX Touch (PerkinElmer, Waltham, MA). Libraries were prepared starting from 500 ng of total RNA, using the TruSeq Stranded mRNA Library Prep Kit (Illumina) and following the manufacturer’s instructions. Samples underwent a paired-end 75 bp sequencing using a NextSeq 500 platform (Illumina).

### Data Analysis

Sequencing reads were mapped to the human genome (hg19) using STAR (version 2.5.2). Transcript quantification from mapped reads was performed using HTSeq-count (version 0.6.1p1) and the human transcripts annotations from Ensembl database (GRCh37 version). Differential expression analysis was carried out using R and the DESeq2 package, considering an FDR<0.1 as threshold.

The enrichment analysis was performed using the Enrichr online tool (Chen et al., 2013), the MSigDB Hallmark pathway, and the PheWeb databases. The co-expression of genes was evaluated starting from transcriptome data and the Coseq R package (Rau and Maugis-Rabusseau, 2018). We applied a centered log ratio (CLR) transformation to count data before fitting a Gaussian Mixture Model, in order to identify gene clusters. For the enrichment analysis we selected only those genes that were attributed to a cluster with a conditional probability>0.9. For each cluster, the average expression profile of all the genes belonging to the cluster was compared between affected and non-affected MZ twins by the Wilcoxon rank sum test.

The estimation of the cellular composition of the samples was performed using the immunedeconv R package, and the quantiseq method (Sturm et al., 2020).

### Quantitative Real-Time Polymerase Chain Reaction

Quantitative RT-PCR was performed following the protocol previously described (Vecellio et al., 2016). RT-PCR was performed in triplicate and the 2−ΔCt method was used to calculate the expression of SMAD3, BRG1 and SNX25 relative to *β*-Actin used as housekeeping gene (ID Assay qHsaCED0036269, Bio-Rad Laboratories, Kidlington, United Kingdom).

Forward and reverse (for and rev) primers are listed below:

SMAD3 for 5′- CAT​CGA​GCC​CCA​GAG​CAA​TA_-3’; SMAD3 rev: 5′- GTG​GTT​CAT​CTG​GTG​GTC​ACT_3’; BRG1 for: 5′- AGT​GCT​GCT​GTT​CTG​CCA​AAT - 3’; BRG1 Rev: 5′-GGC​TCG​TTG​AAG​GTT​TTC​AG -3’.

Primers for SNX25 (for 5′- CCG​TTG​TTC​TCG​TGC​GTT​AA_3′) and (rev: 5′- CCC​ACC​TCG​TTT​ACC​ACT​CG-_3′) were derived from Kato L, et al. Nonimmunoglobulin target loci of activation-induced cytidine deaminase (AID) share unique features with immunoglobulin genes, *Proceedings of the National Academy of Sciences*, 2012, 201120791; DOI: 10.1073/pnas.1120791109.

### Statistical Analysis

We performed one sample *t*-test to determine statistically significance in RT-PCR and MeDIP followed by qPCR experiments. Significance values were set to *p* < 0.05. In Figure 6 and related text, data are represented as mean ± SEM.

### Patient and Public Involvement

It was not appropriate or possible to involve patients or the public in the design, or conduct, or reporting, or dissemination plans of our research.

## Results

### Pso/PsA-Associated Whole Blood Methylome Study

**FIGURE 1:**
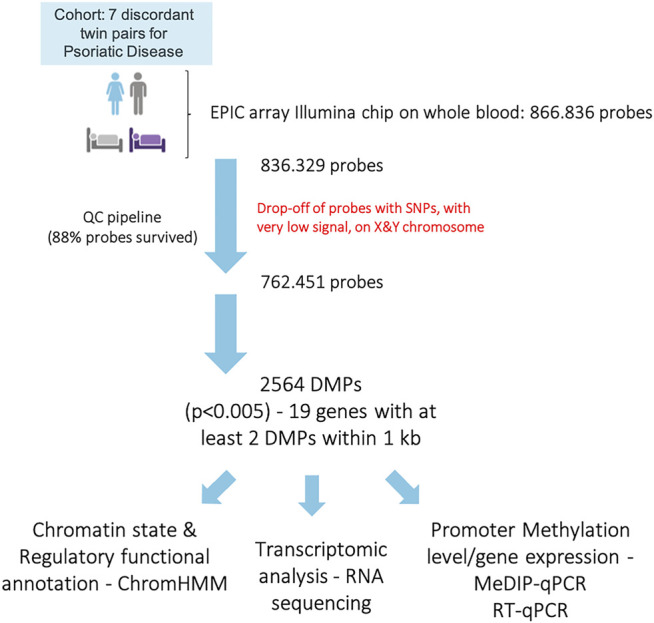
Schematic workflow of the study and analysis conducted.

The genome-wide DNA methylation profile was investigated using the Illumina Inflnium MethylationEPIC BeadChips (EPIC arrays). The general demographic and clinical characteristics of the MZ twins for which methylation data were available are illustrated in Table 1. After performing the quality check and the exclusion of low-quality and unreliable probes, sites that overlap with SNPs, probes with a very low signal and those located on the X and Y chromosomes, methylation data for 762,451 sites were obtained. The experimental workflow and the analyses performed are shown in Figure 1.

**TABLE 1:** Main demographic and clinical features of the 7 pairs of MZ twins discordant for Pso or PsA.

	Pso/PsA affected MZ co-twins (*n* = 7)	Non-affected MZ co-twins (*n* = 7)
Median age, years (range)	44 (25–68)	44 (25–68)
Female/male	1/6	1/6
Pso only	4	—
PsA only	2	—
Pso + PsA	1	—
Family history for psoriatic disease	4	4
Current smoker	2	2
Former smoker	1	2
Years of co-living (average)	25	25

### Identification of DMPs Between Psoriatic Disease Affected and Non-affected Twins

**FIGURE 2:**
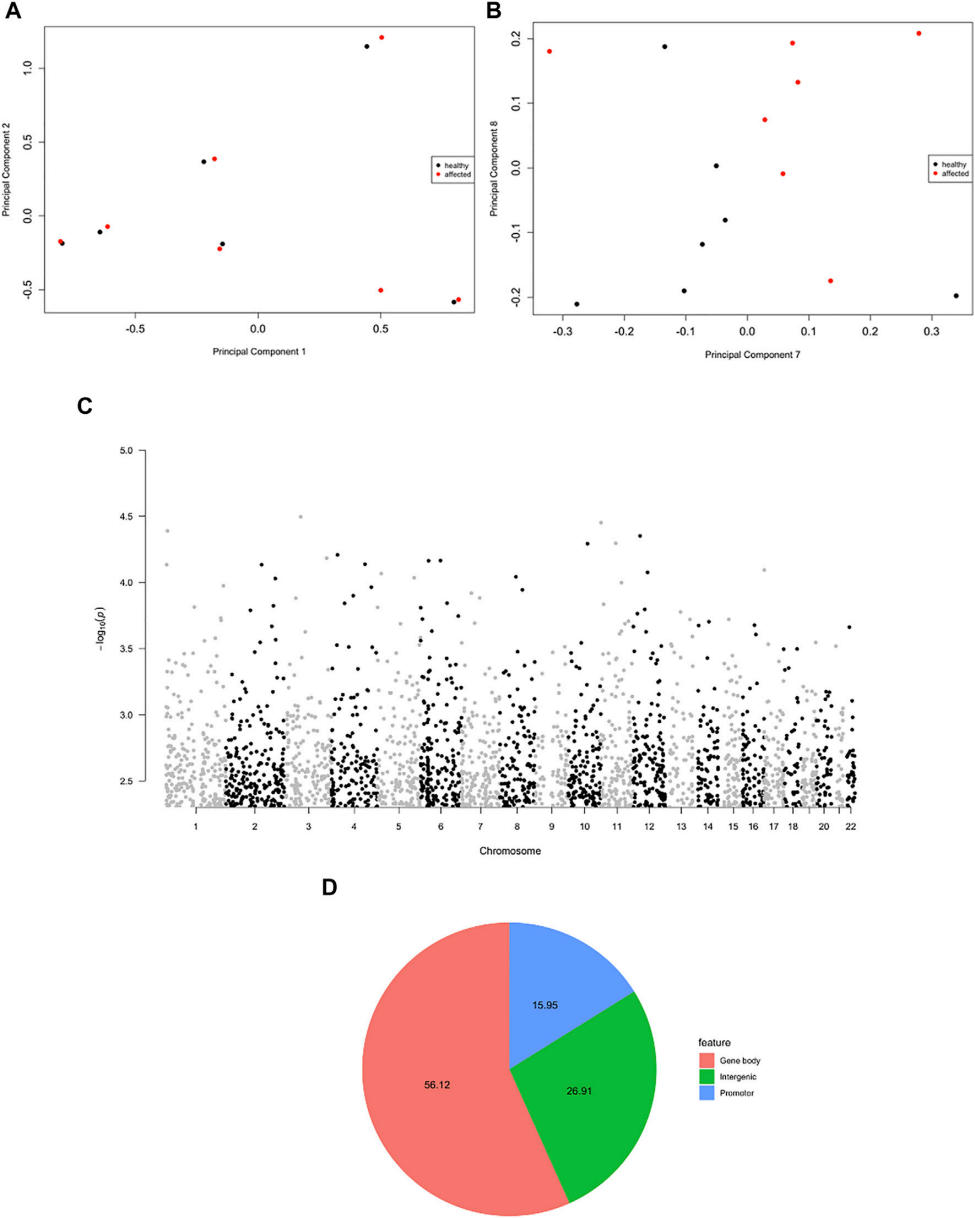
Evidence for psoriatic disease-associated differential DNA methylation in whole blood MZ twins. **(A,B)** A multi-dimensional scale (MDS) plot of all samples is shown, considering the first two components **(A)** and the dimensions seven and eight **(B)**. **(C)** Manhattan plot showing the distribution of the significant DMPs (*p* < 0.005) in the genome. **(D)** Pie chart showing the genomic localization of the DMPs.

The principal component analysis (PCA) highlighted the high similarity between MZ twins (PC1 vs PC2, Figure 2A). However, when higher components were considered, a clear cluster separation between affected and non-affected twins emerged, suggesting a different methylation pattern related to the disease status (PC7 vs PC8, Figure 2B). The statistical analysis did not demonstrate genome-wide significant probes, but when a more stringent filter was applied to the analysis (*p* < 0.005), 2,564 DMPs were observed (Figure 2C), mapping to 1,703 genes (Figure 2D), in the majority of cases at the level of gene bodies (56%), less frequently in intergenic (27%) and promoter regions (16%), in line with the probe distribution in the array, where 51% of probes map to gene bodies, 29% to intergenic and 20% to promoter regions.

**FIGURE 3:**
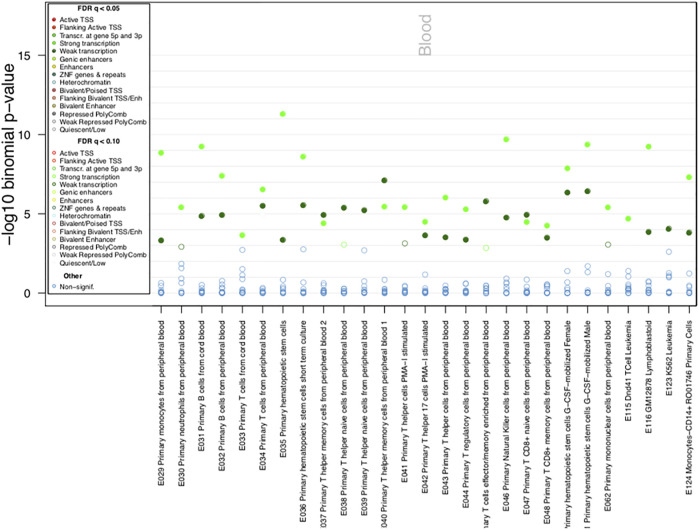
ChromHMM Functional annotation of identified DMPs. Chromatin state enrichment in blood cells as calculated by eFORGE software.

When evaluating whether the Pso/PsA-associated DMPs reside in genomic regulatory regions involved in transcriptional regulation (such as gene promoters, enhancers, transcriptional start sites, specific histone modifications) in different cell types and tissues we observed that the Pso/PsA-associated CpGs were enriched in H3K36Me3 specific histone modifications, especially in blood cells, such as monocytes, lymphocytes, NK cells and hematopoietic stem cells (FDR<0.01, Supplementary Figure S1A). To predict disease-relevant cell types, we investigated the ChromHMM state, which was suggestive of either strong or weak gene transcription overlapping associated DMPs in blood cells (Figure 3) and epithelial fibroblasts (Supplementary Figure S1B) among others (FDR<0.05, *p* < 10^−10^).

**FIGURE 4:**
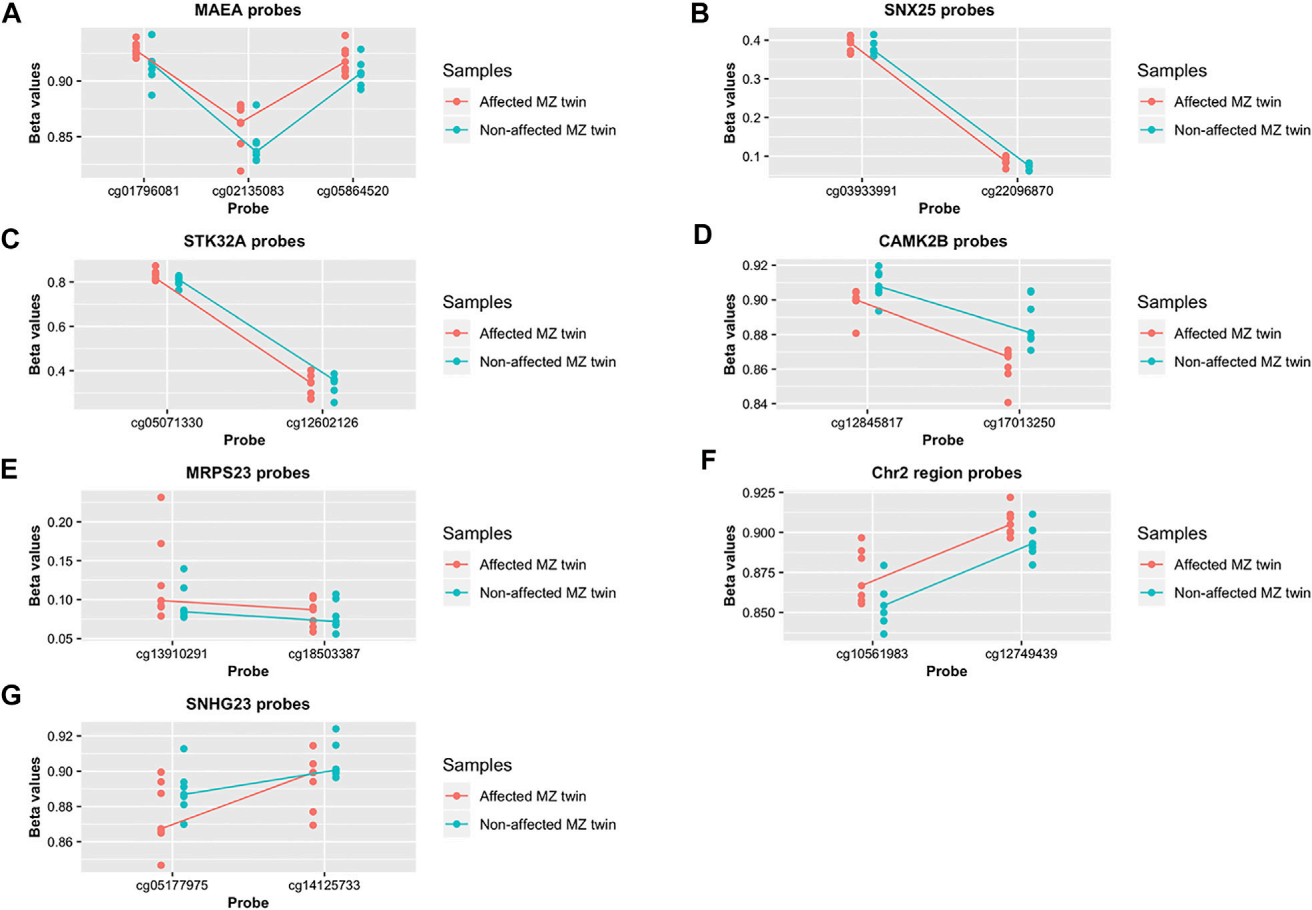
Differential methylation pattern identified in whole blood psoriatic disease MZ twins **(A–G)**. Methylation level of the DMPs localized in the 7 regions characterized by concordant *β*-value variation. Data are presented as a scatterplot, including the individual data points (for the 7 monozygotic twin pairs) that represent the beta values of the significant DMPs. The lines connect the median methylation value of each CpG site, in affected and healthy twins separately (represented in pink and blue, respectively).

We found 19 regions with at least two DMPs within 1 kb, significant within-pair Δ*β*-values and a suggestive *p* < 5 × 10^−3^, as shown in Figure 2C. The 19 regions associated with the identified DMPs are listed in the Supplementary Table S1 and include SF3B1, MAEA, STK32A, CUX1, NUBPL, CDC42BPB, SMAD3, SPNS3, SMARCA4, ARHGEF3, SNX25, EYA4/TARID, CAMK2B, NGDN, SECISBP2L, IGFALS, and MRPS23 genes and one non-coding RNA, SNHG23 as well as a region localized on chromosome 2, where no nearby genes are annotated. Seven of the 19 regions are characterized by a concordant *β*-value variation in all the probes that are distributed in the region (either increased or decreased methylation levels in patients compared to controls) as shown by the scatterplots in Figures 4A–G.

### Psoriatic Disease MZ Twins Have a Distinct Transcriptomic Profile

The RNA sequencing analysis, performed on the same cohort of MZ twins didn’t highlight differentially expressed genes surviving the multiple testing correction. Nonetheless, we performed an enrichment analysis selecting those genes characterized by a significant unadjusted *p*-value (*N* = 662). Very interestingly, when considering deregulated genes, we observed an enrichment in the pathways related to “oxidative phosphorylation,” “inflammatory response,” and “MYC targets” (Supplementary Figure S2, Supplementary Table S2).

**FIGURE 5:**
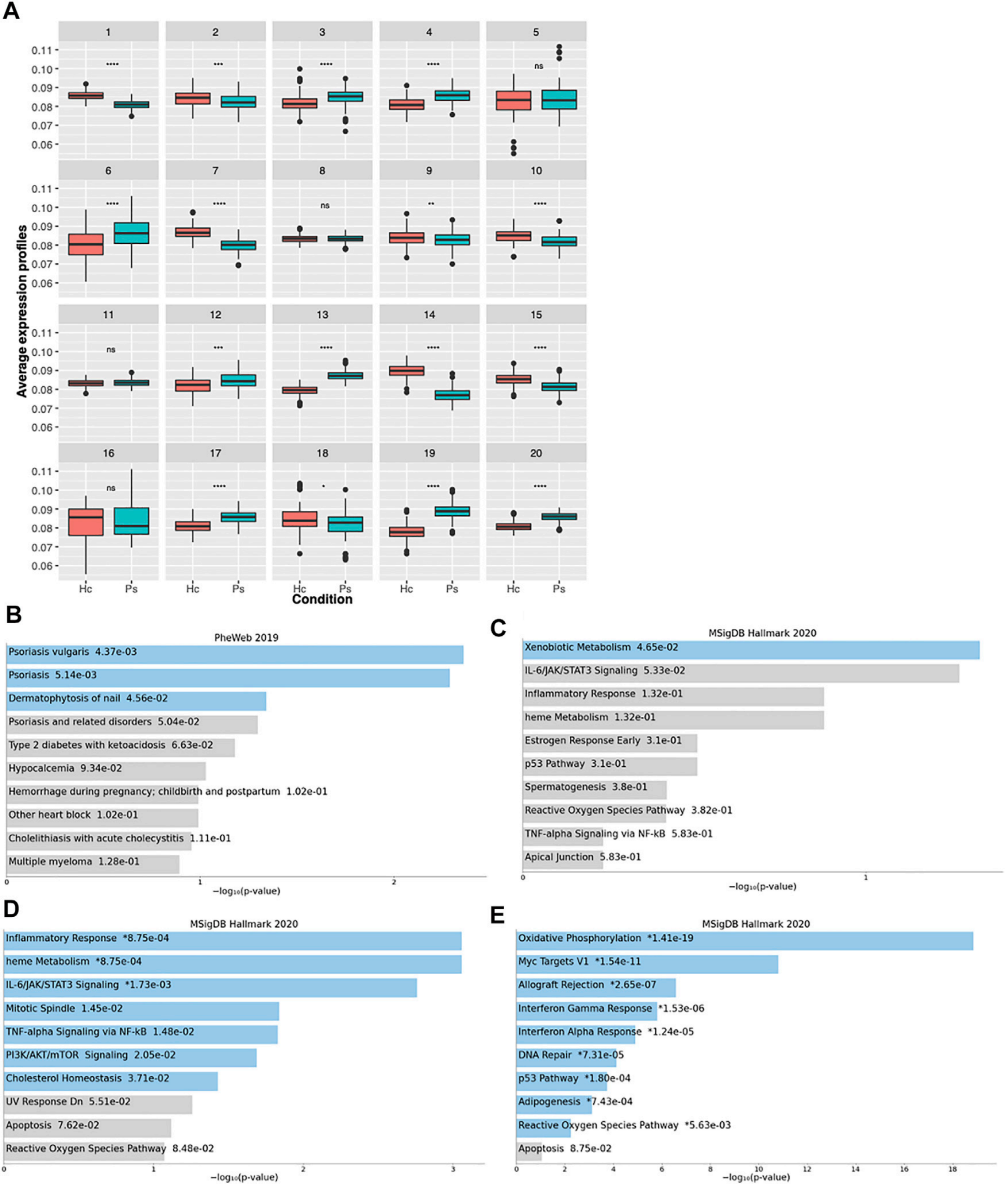
Transcriptional landscape of psoriatic disease-affected MZ twins. **(A)** Boxplots indicating the clusters identified by Coseq2 software, characterized by co-expressed genes. For each cluster, the average expression profile of all the genes belonging to the cluster is shown in affected and non-affected MZ twins. The *p*-values, as calculated by the Wilcoxon rank sum test are displayed at the top of each boxplot. *p < 0.05, **p < 0.01, ***p < 0.001, ****p < 0.0001, ns not significant. Ps: Pso/PsA MZ twin, Hc: unaffected twin. **(B–E)** The bar charts show the top 10 enriched terms in the chosen library (MSig DB Hallmark or PheWas), along with their corresponding *p*-values, for clusters 19 **(B,C)**, 13 **(D)**, 14 **(E)**. Colored bars correspond to terms with significant *p*-values (<0.05). An asterisk (*) next to a *p*-value indicates the term also has a significant adjusted *p*-value (<0.05).

As a next step, we searched for gene clusters that were co-expressed with a different profile between discordant MZ twins. The analysis identified 20 clusters (Figure 5A), the majority showing a statistically significant difference in the average expression profiles between the two groups. We ranked the clusters based on the average expression ratio between affected patients and controls, and we selected the most dysregulated three (clusters 14, 19, 13) to perform an enrichment analysis considering the genes included in each cluster. Interestingly, cluster 19 genes, characterized by a higher average expression profile in affected MZ twins (average expression profile ratio: 1.14, Wilcoxon *p* < 2.2e-16), were associated to “Psoriasis vulgaris” and “Psoriasis” in the PheWeb database (Figure 5B). Moreover, the same set of genes showed a trend for enrichment in the “IL-6/JAK/STAT3” pathway (Figure 5C), a signaling axis that has been implicated in psoriasis (Andrés et al., 2013). Cluster 13, also showing a higher expression in patients compared to controls (average expression profile ratio: 1.1, Wilcoxon *p* < 2.2e-16), confirmed the enrichment in genes involved in the “IL-6/JAK/STAT3” pathway, as well as in “inflammatory response” and “TNF-α signaling pathway” (Figure 5D). Cluster 14, characterized by a higher expression in controls (average expression profile ratio: 0.86, Wilcoxon *p* < 2.2e-16), was instead mainly enriched in “oxidative phosphorylation” pathway (Figure 5E). Finally, we evaluated through a deconvolution method, the cellular composition of the RNAseq samples, but no significant differences were observed between affected and non-affected twins (data not shown).

### Evidence for SNX25 Deregulation in Affected MZ Twins

**FIGURE 6:**
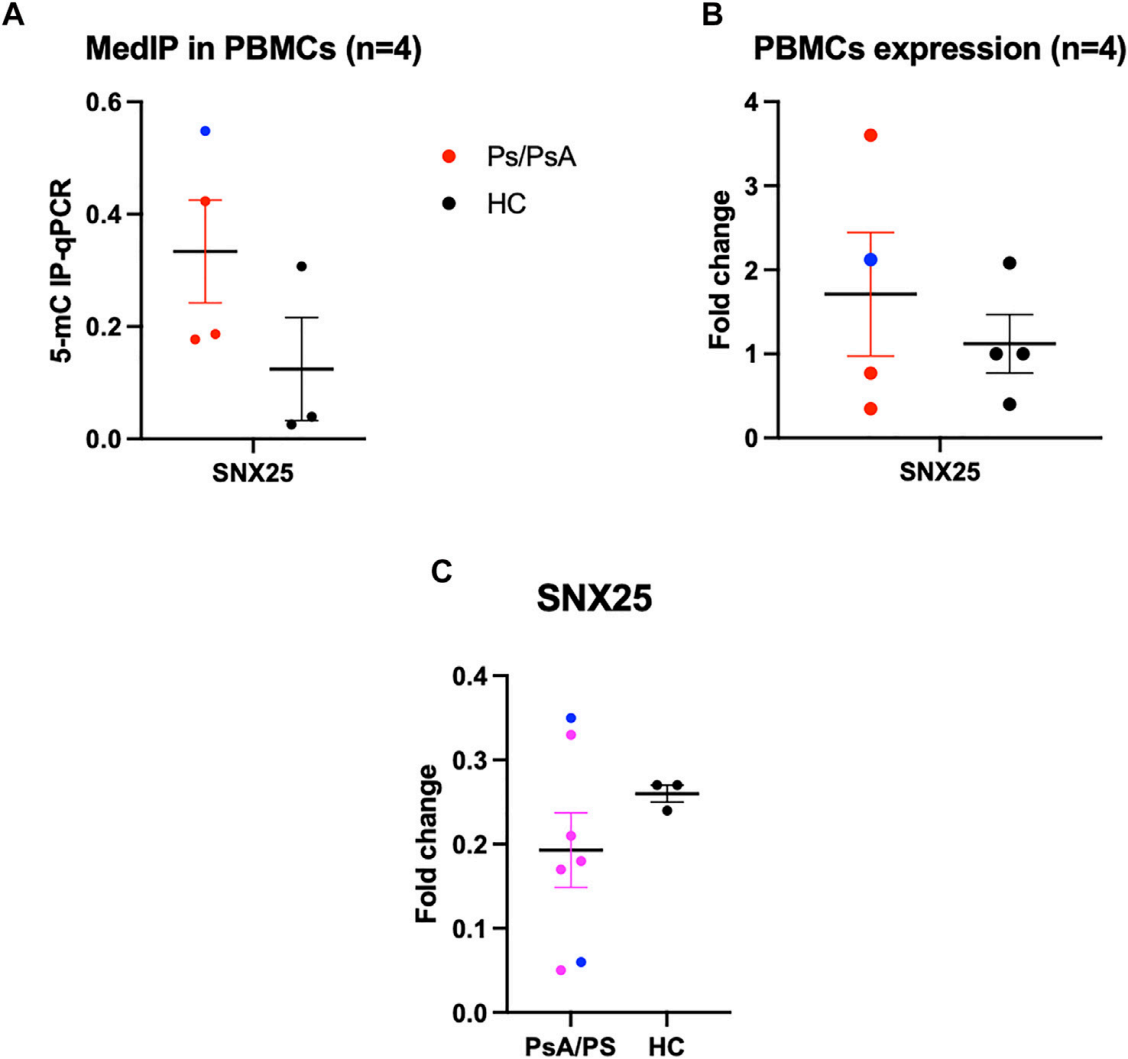
Functional validation of SNX25 gene. **(A)** MeDIp of SNX25 promoter in PsA Twins. **(B)** mRNA expression evaluated with qPCR in PsA MZ Twins. **(C)** SNX25 expression evaluated in a selected cohort of Pso/PsA patients (*n* = 7) vs healthy volunteers (*n* = 3). Blue dots show PsA patients, red dots Pso patients.

Among the differentially methylated regions, only SNX25 resulted also nominally deregulated in MZ twins’ transcriptome data. This gene is involved in the TGF-β pathway and potentially relevant in Pso and PsA pathophysiology. The observed downregulation (logFC = -0.48, *p* = 0.02) is concordant with the methylation profile, with affected MZ twins showing higher methylation levels (Figure 4B). To confirm this result, a quantitative PCR specific for SNX25 promoter was carried out after immunoprecipitation of the methylated DNA in PBMCs of MZ twins. The analysis showed the same trend, although not significant, observed in the methylation array (Figure 6A). No significant differences were observed in the PBMCs’ SNX25 expression of affected and healthy twins (Figure 6B). However, when SNX25 expression was tested in PBMCs of an independent cohort of 7 patients with Pso/PsA and 3 healthy controls the same trend observed in RNAseq data was present (Figure 6C).

### SMARCA4/BRG1 and SMAD3 Show Methylation Differences with Discordant *β*-Value Variation

**FIGURE 7:**
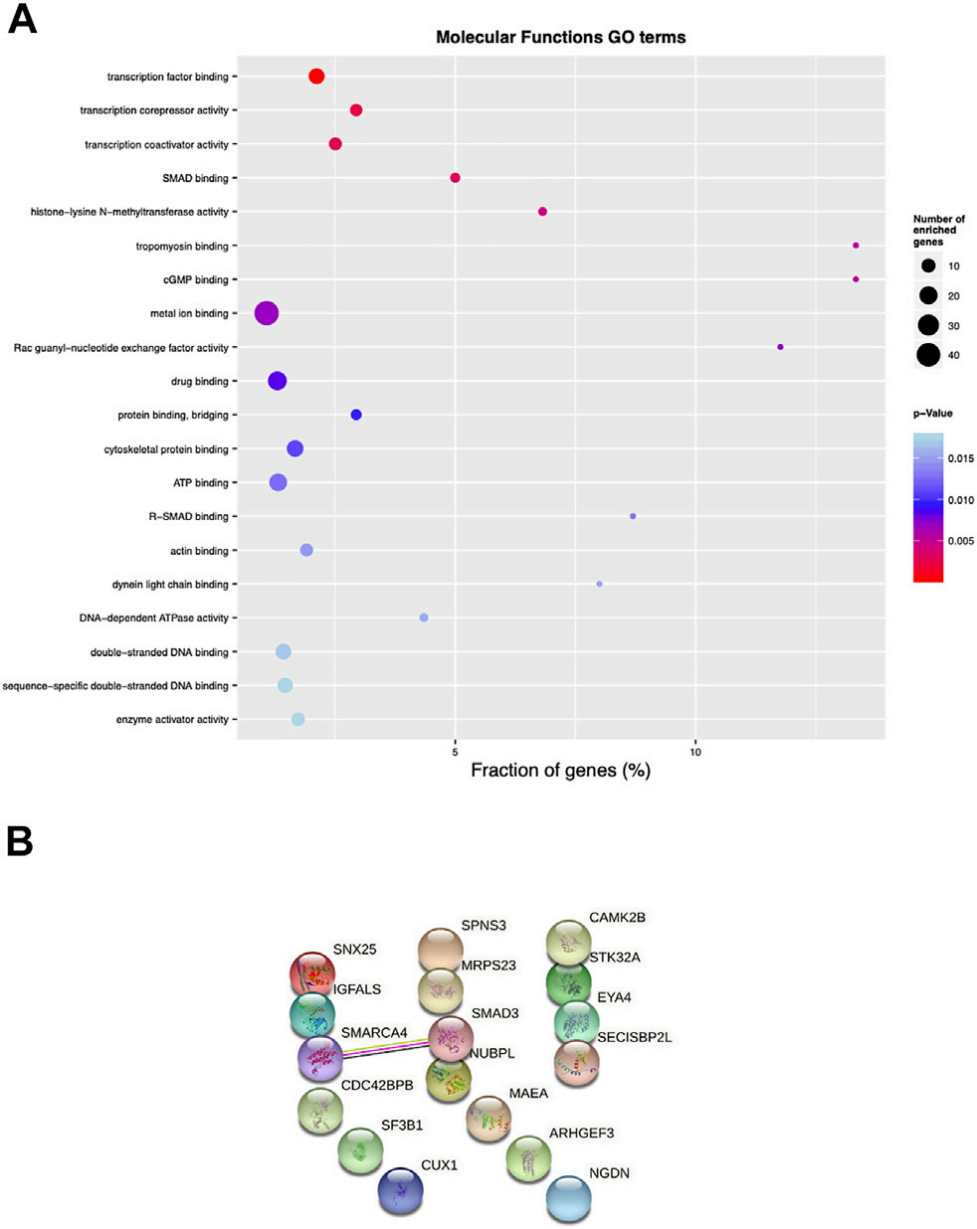
Pathway enrichment and protein-protein interaction analysis of psoriatic disease MZ twins. **(A)** Top 25 differentially enriched GO terms identified by the “topGO” Bioconductor tool, using the “molecular functions” database, ordered by elimFisher *p* value. The width of the dots indicates the percentage of the enriched genes out of the total number of genes belonging to each term. **(B)** protein-protein interaction analysis evaluated with stringdb (https://string-db.org).

A Gene Ontology (GO) analysis of DMPs associated to the 19 regions showed an enrichment in transcription factor binding, transcription corepressor and transcription coactivator activity, SMAD binding and histone -lysine-N-methyltransferase activity (*p* < 0.005) (Figure 7A).

Among the 19 genes identified with the Infinium Methylation EPIC array, we found significant interactions for SMARCA4/BRG1 (transcription activator BRG1, brahma-related gene-1) and SMAD3 (mothers against decapentaplegic homolog 3), by performing protein-protein interaction analysis (String v11.2 database; www.string-db.org) (Figure 7B). The BRG1/SMAD3 interaction was indeed already demonstrated in the literature: immunoprecipitation from cells overexpressing FLAG epitope-tagged BRG1 and HA-tagged SMAD proteins showed that BRG1 is a direct interactor of SMAD3 (Xi et al., 2008). Based on this observation, we further analyzed the methylation status of these two genes. Methylation scatterplots showed a significantly different methylation level of the probes (*p* < 0.005) localized in the SMAD3 and SMARCA4 genes, in affected and healthy twins separately, represented in pink and blue, respectively (Supplementary Figures S3A,B) although with discordant *β*-value variation in the CG probes. We interrogated the promoter of SMARCA4/BRG1 and SMAD3 to evaluate any change in the level of methylation in whole PBMCs from affected and healthy twins. Immunoprecipitation of 5′-methylcytosine antibody followed by quantitative PCR was performed with specific primers for SMARCA4/BRG1 and SMAD3, but we could not identify significant differences between affected and healthy twins (*n* = 4 twin couples, Supplementary Figure S3C). Further, no changes were found in SMARCA4/BRG1 and SMAD3 mRNA expression in PBMCs (Supplementary Figure S3D). We also evaluated the mRNA expression of SMAD3 and SMARCA4/BRG1 in the selected control cohort of 7 patients with Pso/PsA and 3 healthy controls, confirming no significant changes (Supplementary Figures S3E,F).

## Discussion

Epigenetic changes have widely been proposed to be crucial in the pathogenesis of complex diseases (Petronis, 2010) and, more specifically, DNA methylation is linked to different immune-mediated disorders, from rheumatoid arthritis to multiple sclerosis (Liu et al., 2013; Dick et al., 2014). While epigenetic changes are ideal mechanisms to determine disease discordance in MZ twins, translating EWAS data into a quantifiable epigenetic difference and mechanistic understanding for complex diseases like Pso and PsA (herein cumulatively identified as psoriatic disease) is challenging, particularly in the absence of disease biomarkers. We performed the first genome-wide integrative approach to identify differential DNA methylation in whole blood from a selected cohort of MZ twins discordant for psoriatic disease, similar to what has been performed in other complex diseases (Javierre et al., 2010; Rakyan et al., 2011).

Our EWAS approach identified distinct DNA methylation patterns at specific DMPs differentiating affected and healthy twins, mapping to the gene body of 1,703 genes while we did not observe genome-wide significant differences [*p* < 3.6 × 10^−8^ (Saffari et al., 2018)]. The ENCODE and Blueprint regulatory annotations showed an enrichment for H3K36me3 histone mark overlapping the significant DMPs. DNA promoters methylation is largely known for its repressive effect on transcription initiation of protein‐coding genes, non‐coding RNAs, or transposon repeats (Teissandier and Bourc’his, 2017), while the function of intragenic methylation remains unclear (Baubec et al., 2015) and it may be involved in the modulation of alternative splicing, in exon definition and recognition (Maunakea et al., 2013). The commonly accepted dogma where methylation negatively correlates with gene expression as and vice versa, not always hold as, in different cancers, within promoter regions it has been observed a positive correlation between methylation and gene expression (Chatterjee and Vinson, 2012; Kim et al., 2016; Spainhour et al., 2019). H3K36me3 is required for preventing spurious transcription initiation stemming from transposon fragments or cryptic promoters, preserving transcription process to canonical promoters (Verma et al., 2018). It has been recently demonstrated that H3K36me3 may act as a docking site for DNMT3B (DNA Methyltransferase 3 Beta), and together with reduced H3K4me3, altogether they repress the default activity of intragenic CpG islands and act as transcriptional promoters (Baubec et al., 2015). Further, the enrichment of methylated cytosines in regulatory regions that we identified in blood cell subsets suggests a dysregulation in psoriatic disease, which might be cell-type specific.

The enrichment analysis performed on transcriptome data suggested a significant role of “oxidative phosphorylation,” “inflammatory response” and “myc targets” pathways in disease susceptibility. Indeed, these pathways have been already associated to autoimmune disorders, and also to Pso/PsA. Regarding the “oxidative phosphorylation” pathway, it was observed that there is a direct link between dysregulated glucose metabolism in lymphocytes and autoimmunity (Bantug et al., 2018). In fact, enhanced glycolytic activity is a common feature of pro‐inflammatory effector CD4 lymphocytes such as T helper (Th) 1 and Th17, cells characterized by a Warburg‐like glycolytic metabolism, since they require aerobic glycolysis for their differentiation and effector functions. On the other hand, Regulatory T (Treg) cells, which suppress inflammatory responses and promote tolerance, after the initial activation, are characterized by an enhanced oxidative metabolism (Kornberg, 2020). The co-expression analysis again evidenced the dysregulation of this pathway, since we found an enrichment of the “oxidative respiration” pathway in cluster 14, characterized by a higher gene expression in non-affected MZ twins. It is worth mentioning that one of the top dysregulated genes in the transcriptome data is PDK4, coding for Pyruvate Dehydrogenase Kinase 4, a protein implicated in glucose metabolism. The gene was upregulated in affected MZ twins (logFC = 0.64, unadjusted *p* = 1.1*10^−4^). A recent study evaluated the effect of PDK4-deficiency on the development of experimental autoimmune encephalomyelitis, the mouse model of multiple sclerosis, another autoimmune disorder, induced by pathogenic Th17 cells. Very interestingly, knock-out mice developed a less severe disease and showed a decrease in Th17 cells and an increased infiltration of Foxp3+ Tregs in the central nervous system (Allon Wagner et al., 2020). All together these results suggest that in affected MZ twins there might be a higher activation state of pro-inflammatory, and hence disease-supporting, Th lymphocytes.

Also MYC is one of the key players that coordinate metabolic reprogramming and activity in immune cells (Gnanaprakasam and Wang, 2017), which play a pivotal role in the development of inflammation and autoimmunity.

The finding that seven genomic regions are characterized by a concordant *β*-value variation in all the probes distributed, either with increased or decreased methylation level between affected and healthy twins, is of primary importance in order to define a specific methylation signature associated with psoriatic disease. Among these seven genes, SNX25, a negative regulator of TGFβ pathway and intracellular trafficking, was previously found associated among the differentially methylated sites between psoriatic and normal skin (Verma et al., 2018). Interestingly, transcriptome data pointed to a deregulation of this gene in affected MZ twins. Further analysis performed to replicate the results showed a trend, although not significant, both in methylation status and in expression level of the gene. Additional analyses are needed on a larger cohort of patients to confirm these results.

A small number of the 19 genes with at least two DMPs within 1 kb of distance and significant within-pair Δβ-values was found significantly associated with psoriatic disease. Among those, SMAD3, which is involved in the downstream signaling pathway of TGF-β, is associated with BRG1 to mediate TGF-*β*-induced transcriptional regulation at multiple genes, by means of chromatin remodeling and gene expression regulation. Although the methylation level of the CpG probes located in SMAD3 and SMARCA4/BRG1 was significant, this did not follow a consistent trend. Further, despite no significant changes in RNA expression were observed, a possible contribution of the differential methylation pattern in the definition of the alternative splicing profile of the gene cannot be ruled out.

A few studies evaluated the role of DNA methylation in the disease susceptibility specifically in psoriasis (Gervin et al., 2012; Li et al., 2020) at genome-wide level in immune cells. The most comprehensive one (Gervin et al., 2012), focused on lymphocytes, didn’t disclose any differentially methylated regions between co-twins; however, when gene expression was also considered, different genes were identified. Among the 50 top hits, 7 of them (14%) were found differentially methylated in our dataset. Moreover, GO analysis revealed enrichment in processes associated with “immune response” and “psoriasis.” This is in accordance with our results, since in the co-expression analysis we highlighted an enrichment in genes associated with “inflammatory response,” “IL6/STAT/JAK signaling,” as well as involved in psoriasis. The role of inflammatory response was also evidenced by another genome-wide transcriptome analysis (Rawat et al., 2020) where a consistent, though small, pattern of changes was observed for a set of genes associated with “neutrophil activation” and “inflammation.” Even though all the data point to the involvement of the same pathways in disease susceptibility, the lack of a high degree of overlap in the identified genes can be due to the use of different starting samples.

We are aware of the strengths and limitations of our study. Among the former, the chosen model of MZ twins is a unique setting to investigate complex diseases, as hereditary factors are not the major determinants of immunological functions, as proven in healthy MZ twins (Brodin et al., 2015). Further, using twins can improve the statistical power of a genetic study as it reduces the amount of genetic and/or environmental variability. Conversely, the results obtained from twin studies cannot be universally correlated to the general population, due to lack of randomization (Sahu and Prasuna, 2016). We are aware that our twin’s cohort is heterogenous (see Table 1 for patients’ characteristics), as it includes patients affected by psoriasis, psoriatic arthritis and both manifestations simultaneously. Further, the multitier approach with a small independent cohort of unrelated patients and controls also represents an advantage for our study. Among the study limitations, DNA methylation was measured in peripheral blood cells while epigenetic changes associated with psoriatic disease may be present in specific tissues such as the skin, but the collection of these samples through biopsies is invasive, and the collection of synovial fluid may not be feasible in most of PsA cases when synovitis is not the predominant domain. For this reason, peripheral blood is the best accessible alternative that reflects different pathophysiological pathways and there is high potential clinical utility for any identified blood-derived epigenetic disease marker. Nevertheless, we are aware that the results we obtained in whole blood, could represent a limitation, as the observed changes cannot be associated with a specific cell type (i.e., T-cells, monocytes).

In conclusion, our genome-wide study on the unique model of MZ twins demonstrates high similarity in whole blood-based methylomes of psoriatic disease-discordant samples. However, we identified DMPs specific for psoriatic disease and few candidate loci that warrant to be further evaluated. The evaluation of the causal relation between genetic variants, biomarker levels and DNA methylation is becoming of undisputed importance. Their interplay is relevant to define plausible biological pathways in psoriatic disease that are regulated by epigenetic mechanisms.

## Data Availability

The datasets presented in this study can be found in online repositories. The names of the repository/repositories and accession number(s) can be found below: GSE186713 for methylation data, GSE186724 for RNA-seq data.
