# Supplementary material for: DNA Methylation Signature in Monozygotic Twins Discordant for Psoriatic Disease
Source: Front Cell Dev Biol. 2021 Nov 24;9:778677. doi: 10.3389/fcell.2021.778677 (PMC8653905; doi:10.3389/fcell.2021.778677)
Supplement: Supplementary file 1 [file DataSheet1.PDF]

Suppl Fig 1

A

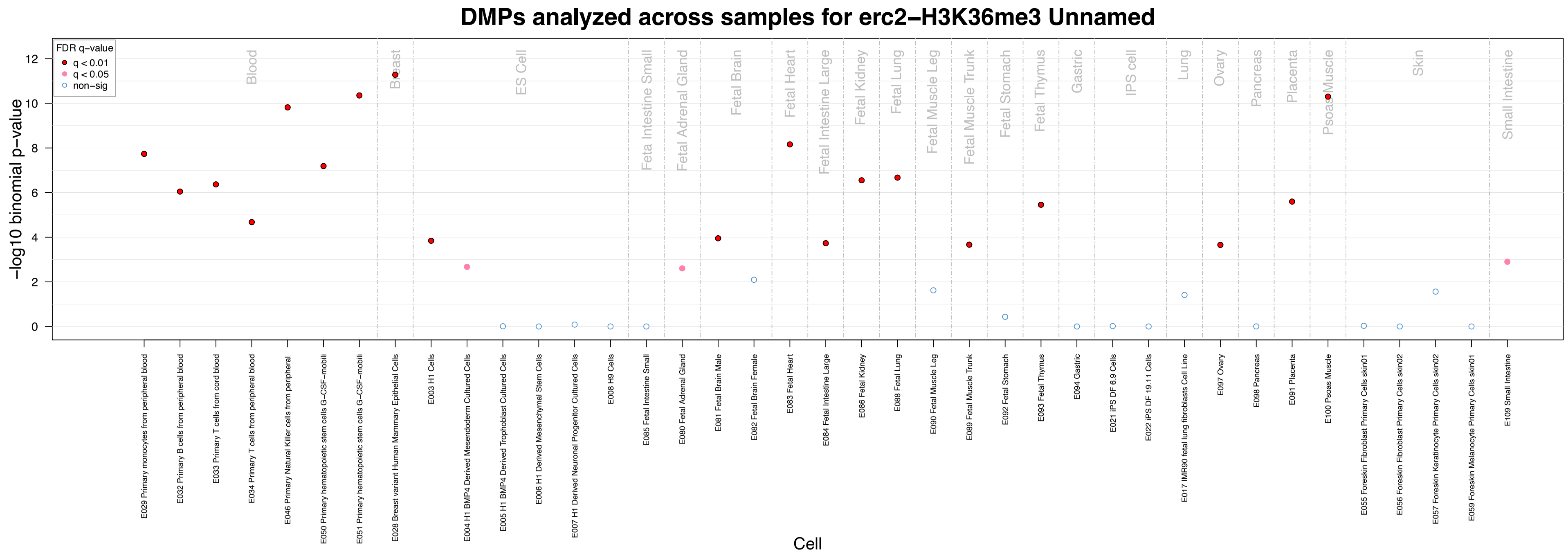

B

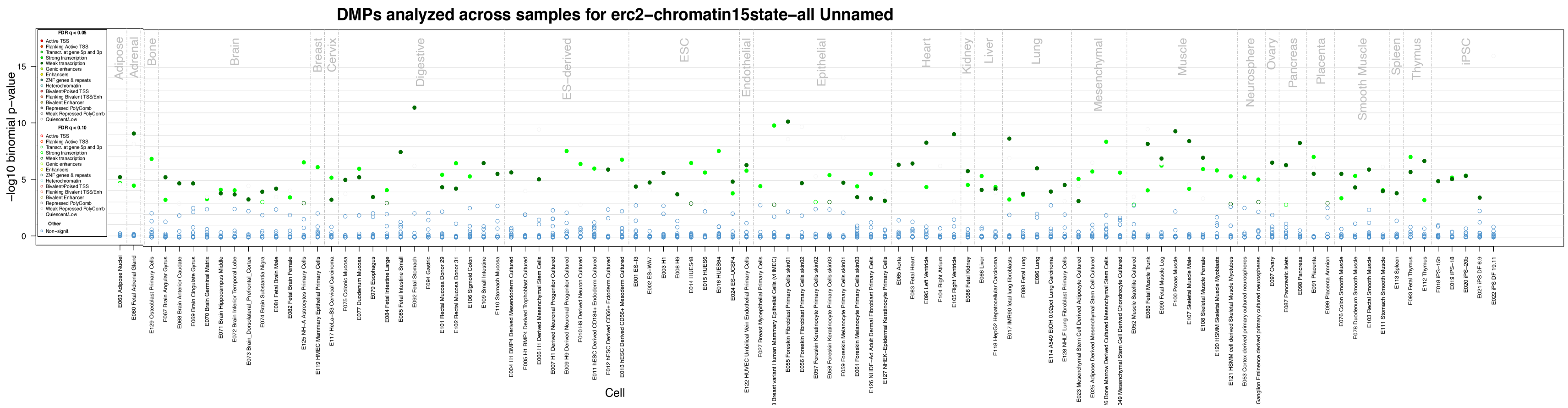

Suppl Fig 2

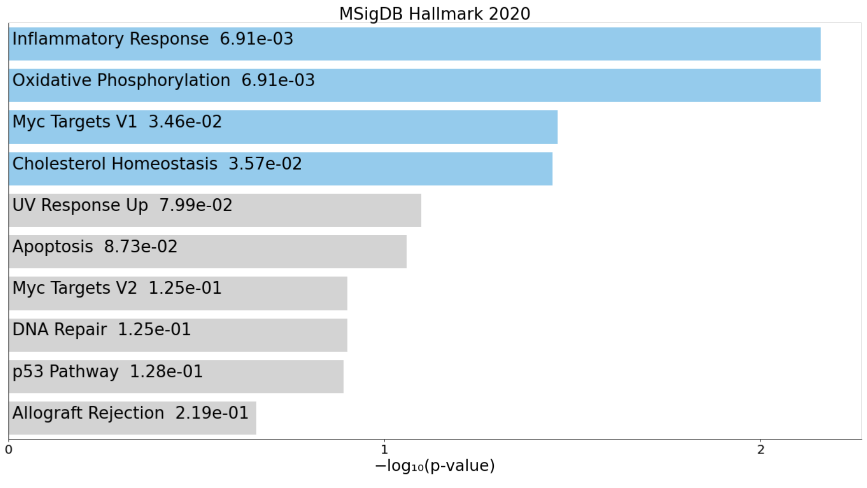
MSigDB Hallmark 2020
Inflammatory Response 6.91e-03
Oxidative Phosphorylation 6.91e-03
Myc Targets V1 3.46e-02
Cholesterol Homeostasis 3.57e-02
UV Response Up 7.99e-02
Apoptosis 8.73e-02
Myc Targets V2 1.25e-01
DNA Repair 1.25e-01
p53 Pathway 1.28e-01
Allograft Rejection 2.19e-01
0
1
2
−log10(p-value)

Suppl Fig.3

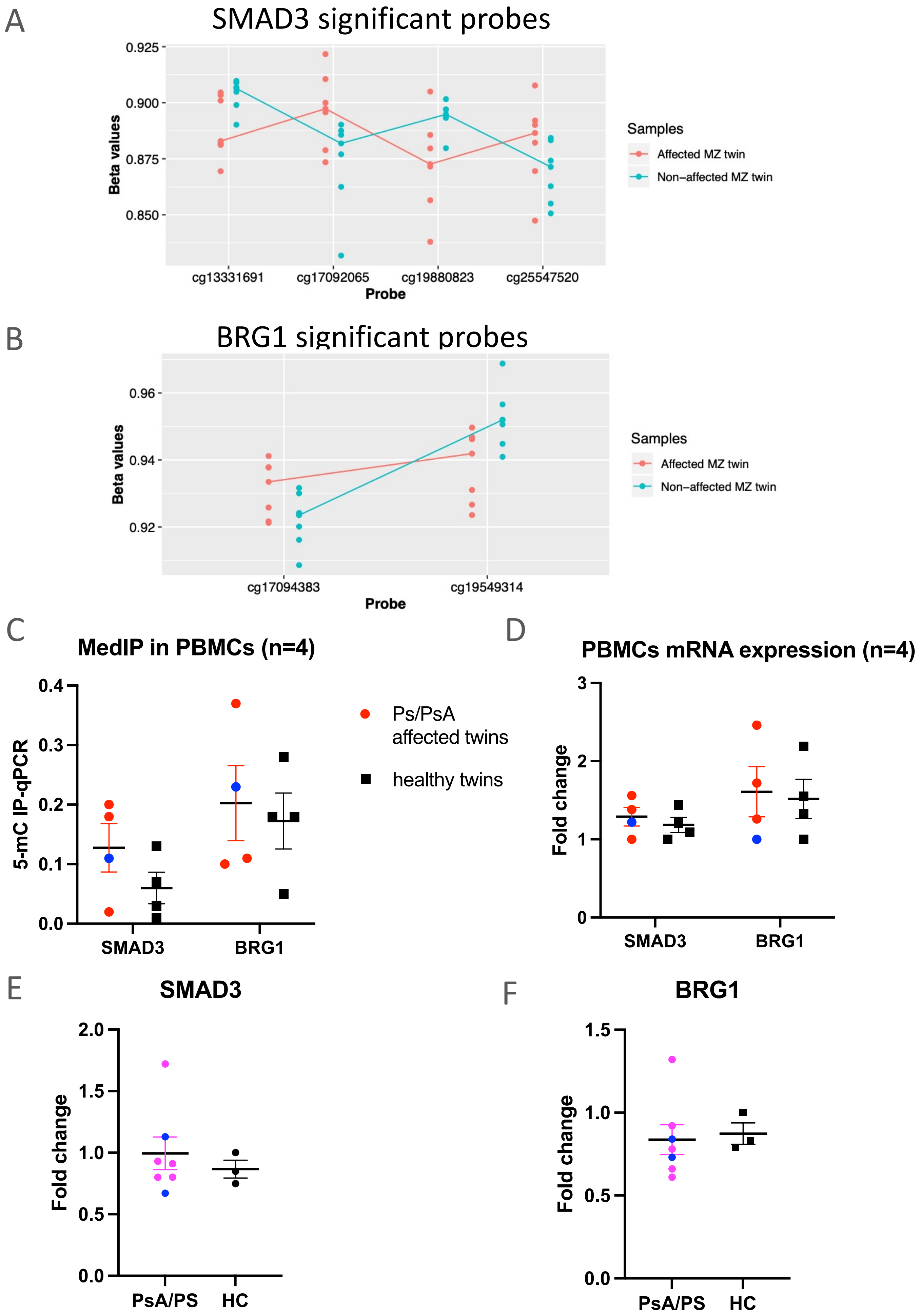
A
SMAD3 significant probes
Beta values
0.925
0.900
0.875
0.850
cg13331691
cg17092065
cg19880823
cg25547520
Probe
Samples
Affected MZ twin
Non–affected MZ twin
B
BRG1 significant probes
Beta values
0.96
0.94
0.92
cg17094383
cg19549314
Probe
Samples
Affected MZ twin
Non–affected MZ twin
C
MedIP in PBMCs (n=4)
5-mC IP-qPCR
0.4
0.3
0.2
0.1
0.0
SMAD3
BRG1
Ps/PsA
affected twins
healthy twins
D
PBMCs mRNA expression (n=4)
Fold change
3
2
1
0
SMAD3
BRG1
E
SMAD3
Fold change
2.0
1.5
1.0
0.5
0.0
PsA/PS
HC
F
BRG1
Fold change
1.5
1.0
0.5
0.0
PsA/PS
HC
